# Bufalin post-transcriptionally suppresses STAT3 to alleviate renal ferroptosis and tubulointerstitial fibrosis in diabetic kidney disease

**DOI:** 10.1080/0886022X.2026.2667591

**Published:** 2026-05-26

**Authors:** Yunyang Qiao, Chen Gao, Jialing Ji, Zuolin Li, Huimin Shi, Ruilian Yan, Jiayue Sun, Yueyi Wang, Qiang Lin, Aiqing Zhang

**Affiliations:** a Department of Pediatrics, the Fourth Affiliated Hospital of Nanjing Medical University, Nanjing, Jiangsu, China; b Department of Pediatrics, Changzhou Seventh People’s Hospital, Changzhou, Jiangsu, China; c Institute of Nephrology, Zhong Da Hospital, Southeast University School of Medicine, Nanjing, Jiangsu, China; d Department of Pediatric Nephrology, the Second Affiliated Hospital of Nanjing Medical University, Nanjing, Jiangsu, China; e The Second Clinical Medical School, Nanjing Medical University, Nanjing, Jiangsu, China; f Department of Nephrology and Immunology, Children’s Hospital of Soochow University, Suzhou, Jiangsu, China

**Keywords:** Diabetic kidney disease, tubulointerstitial fibrosis, ferroptosis, bufalin, signal transducer and activator of transcription 3

## Abstract

The pathological hallmark of diabetic kidney disease (DKD) is tubulointerstitial fibrosis (TIF), which arises from extracellular matrix (ECM) synthesis in renal tubular epithelial cells (RTECs). Bufalin has the potential in delaying the progression of kidney disease, and ferroptosis plays a critical role in this regulatory process. In this study, we investigated the effects of bufalin on DKD models by analyzing the expression of TIF and ferroptosis-related markers and evaluating the impact of ferrostatin-1 (Fer-1) on TIF. Our findings revealed that the treatment of bufalin in *db/db* mice and high glucose (HG)-induced RTECs altered the expression of TIF-related indicators. Ferroptosis was activated during the progression of TIF. Characteristic changes in ferroptosis, including iron overload, increased production of lipid peroxidation products, and decreased mitochondrial cristae, are induced by HG, and the treatment of Fer-1 reversed ferroptosis and further mitigated TIF. Notably, the intervention of bufalin in DKD models exhibited an inhibitory effect on ferroptosis. Network pharmacology analysis identified signal transducer and activator of transcription 3 (STAT3) as a target of bufalin, and knockdown of STAT3 altered the expression of ferroptosis and TIF-related indicators. Molecular docking studies, dual-luciferase reporter assay, and mRNA stability analysis further elucidated the regulation of STAT3 by bufalin and regulatory mechanism. Overall, these results demonstrate that bufalin promotes the degradation of STAT3 mRNA *via* a post-transcriptional mechanism. This downregulation inhibits STAT3-mediated ferroptosis, ultimately alleviating TIF in DKD.

## Introduction

Diabetic kidney disease (DKD) is the leading cause of end-stage renal disease and has become a global public health problem [1–3]. Renal fibrosis is the recognized pathological pathway of DKD, in which tubulointerstitial fibrosis (TIF) is a common pathological change in the progression of chronic kidney disease (CKD) to end-stage renal disease from different etiologies [4–6]. In the pathological basis of DKD, renal tubular epithelial cells (RTECs) are important fibrotic effector cells, and the occurrence of epithelial–mesenchymal transition (EMT) and the deposition of extracellular matrix (ECM) synergistically facilitate the progression of TIF [4,7].

Extracted from the skin and parotid gland of the toad, bufalin is a traditional Chinese medicine that has received increasing attention from clinicians and researchers for its cardiotonic, antitumor, and immunomodulatory effects [8,9]. With respect to its antitumor activity, bufalin has been shown to inhibit the growth of a variety of tumor cells; however, little research has been conducted on its use in treating kidney diseases. A study confirmed that bufalin can inhibit the proliferation of mesangial cells by regulating the cell cycle, suggesting that bufalin may be a potential therapeutic agent for the prevention of mesangial proliferative glomerulonephritis and that its therapeutic potential in CKD needs to be further explored [10].

Here, a series of analyses targeting the target of bufalin revealed that its role in the development of DKD is highly likely to involve the regulation of ferroptosis. Ferroptosis is a novel form of programmed cell death characterized by iron overload, lipid peroxide accumulation, and altered mitochondrial morphology [11]. The essence of ferroptosis lies in the reduction of the activities of glutathione peroxidase 4 (GPX4) and solute carrier family 7 member 11 (SLC7A11), as well as the depletion of glutathione. Meanwhile, the activation of long-chain acyl-CoA synthetase family member 4 (ACSL4) promotes the process of ferroptosis [12–16]. The pathogenesis of renal diseases is closely related to several forms of intrinsic cell death [17,18]. Although ferroptosis was initially identified in cancer cells, a growing number of studies have elucidated the role that ferroptosis plays in renal diseases. The inhibition of ferroptosis attenuates lipid peroxidation, oxidative stress, and cellular injury in various chronic and acute kidney disease models [19–21].

Bufalin attenuates inflammatory responses and oxidative stress and has been shown to be involved in the regulation of cell death, such as pyroptosis [22,23]. The role of bufalin in DKD may involve the regulation of ferroptosis, but this role remains elusive. To elucidate the therapeutic potential of bufalin in TIF associated with DKD, pharmacological inhibition of ferroptosis using ferrostatin-1 (Fer-1) significantly attenuated TIF *in vivo* and *in vitro*, thereby confirming the causal contribution of ferroptosis to DKD progression. Subsequently, we evaluated bufalin’s efficacy and underlying mechanism. Integrated network pharmacology and experimental validation identified signal transducer and activator of transcription 3 (STAT3) as a central molecular target of bufalin. Mechanistically, bufalin promoted the degradation of STAT3 mRNA *via* a post-transcriptional regulatory pathway, leading to downregulation of STAT3 expression and subsequent inhibition of STAT3-mediated ferroptosis. Collectively, these findings position bufalin as a promising STAT3-targeted anti-ferroptotic agent for mitigating TIF in DKD.

## Materials and methods

### Animal model and therapeutic experiments

The *db/db* mouse is a suitable subject for the study of DKD [24]. Male *db/db* mice and *db/m* mice used as animal models of DKD were purchased from GemPharmatech LLC (Nanjing, China). All the mice were raised in an environment with constant temperature, constant oxygen supply, and a 12-h light-dark cycle. All the mice were fed with ordinary food and water and were housed in the animal experimentation center of Nanjing Medical University. Intervention with bufalin (HY-N0877R, MCE, Monmouth Junction, USA) and Fer-1 (HY-100579, MCE, Monmouth Junction, USA) was performed when the mice were 12 weeks old. The mice were grouped into (1) the *db/m* group; (2) the *db/db* group; (3) the *db/db*+dimethyl sulfoxide (DMSO) (HY-Y0320C, MCE, Monmouth Junction, USA) group; (4) the *db/db*+bufalin group; and (5) the *db/db*+Fer-1 group. Under the conditions of fulfilling the 3 R principles for experimental animals and adhering to statistical requirements, each group was set with a sample size of 6. The experimental mice were randomly grouped using the method of generating random numbers by computer. In the *db/db*+bufalin group, bufalin was injected intraperitoneally into *db/db* mice at a dose of 0.1 mg/kg for 14 consecutive days starting at 12 weeks of age [25]. The *db/db*+Fer-1 group was injected intraperitoneally with 1 mg/kg dissolved Fer-1 every 2 days for 8 weeks. Both bufalin and Fer-1 were dissolved in DMSO and further diluted, and a control, *db/db*+DMSO, was used for all interventions.

The *in vivo* experimental design of this study adheres to the principles outlined in the ARRIVE guidelines and has been rigorously validated for reliability across all aspects. All experiments were conducted in compliance with the ethical standards and regulatory requirements approved by the Experimental Animal Welfare Ethics Committee of Nanjing Medical University (Approval No. IACUC-2404054). All procedures strictly adhered to the guidelines outlined in the ‘Guide for the Care and Use of Laboratory Animals’, with the aim of minimizing animal suffering throughout the experimental process. At 20 weeks of age, the mice were anesthetized and sacrificed with sodium barbiturate (100 mg/kg), and blood, urine, and kidney tissues were collected for subsequent experiments.

### Quantification of 24-h urine protein and serum creatinine (SCr)

Urine was collected from all the mice for 24 h, and the urine volume was recorded. The collected urine and blood were centrifuged and the lower sediment was discarded. The supernatant was collected for the measurement of the urine protein content and SCr level according to the instructions of the assay kit (C035-2-1, C011-2-1, Jiancheng, Nanjing, China). The absorbance values of all the wells at 595 nm were determined *via* an enzyme meter. The urinary protein concentration (mg/L) of all samples was calculated *via* the formula, and the 24-h urinary protein value was further calculated from the 24-h urine volume of each mouse. For the assay of SCr, the absorbance values of all the wells were measured twice at 546 nm, during which time the plate was incubated at 37 °C for 5 min, and the amount of SCr (µmol/L) in each sample was calculated according to the formula.

### Sirius red, periodic acid-Schiff (PAS), and Masson staining

The preparation steps for pathologic staining are almost identical. The embedded tissue wax blocks were cut to a thickness of 3 μm, placed on a paddle, and then baked in an oven for at least 2 h. Subsequently, the waxes were fully dewaxed and hydrated *via* xylene and ethanol in a fume hood and washed clean with double-distilled water. The sections were sequentially immersed in staining solution *via* the appropriate pathology staining kit (G1088, G1006, Servicebio, Wuhan, China), and the nuclei were restained if necessary, rinsed well with running water halfway through, soaked again with ethanol and xylene, and sealed with neutral gum, a step that enables dehydration and visualization. After sealing, the slices were examined under a microscope, and images were collected for analysis.

### Immunofluorescence (IF) and immunohistochemistry (IHC)

Sections were heated at 70 °C for at least 2 h, followed by deparaffinization and hydration. The antigen repair solution was prepared, heated to boiling, cooled at room temperature, and then blocked with 5% bovine serum albumin for 2 h. Primary antibodies against fibronectin (FN) (1:300, ab2413, Abcam, Cambridge, UK), anti-collagen I (Col I) (1:100, sc-59772, Santa Cruz, Dallas, USA), vimentin (1:100, BF8006, Affinity, Changzhou, China), and anti-STAT3 (1:100, AF6294, Affinity, Changzhou, China) were prepared, added dropwise to the tissue, and incubated in a wet box overnight. The following day, the sections were washed, and the corresponding secondary antibody was added dropwise. After the nuclei were stained, the sections were dehydrated again and sealed, and images were obtained and analyzed under a confocal microscope (FV1000, Olympus, Tokyo, Japan). The histopathological scores were independently completed by three researchers without being informed of any specific information.

### Cell lines and culture conditions

RTECs were a gift from the Institute of Nephrology, Zhong Da Hospital, Southeast University School of Medicine, and were originally purchased from Pricella Biotechnology Co., Ltd. (CP-M062, Wuhan, China). The cells were cultured for a long period of time and fully acclimatized in normal glucose (5.5 mM) Dulbecco’s modified Eagle’s medium (11885084, Gibco, Rockville, USA) supplemented with 10% fetal bovine serum. Glucose powder was used to dissolve and filter the HG solution to serve as the HG group (30 mM), whereas the mannitol group (5.5 mM glucose + 24.5 mM mannitol) was used to exclude the effects of high osmolarity of the solution. Similar to the *in vivo* experiments, further interventions for the DKD cell model were divided into the HG+bufalin group, the HG+Fer-1 group, and the corresponding DMSO group. All interventions were set for 24 h.

### Cell viability assay

The cell counting kit-8 (CCK-8) (BMU106, Abbkine, Wuhan, China) was used to determine the most appropriate intervention protocol for HG and to evaluate the cytotoxic effect of bufalin. RTECs were seeded at a density of 500 cells per well in a 96-well microplate. After treating the cells, they were washed with phosphate-buffered saline, and then, 100 μL of the culture medium containing 10 μL of CCK-8 solution was added. Finally, the absorbance was measured at a wavelength of 450 nm to assess cell viability.

### Cell transfection

The STAT3 small-interfering RNA (siRNA) and the corresponding negative control (NC) were provided by Santa Cruz (sc-29494, Dallas, USA). The transfection mixture was prepared at the final concentration of 10 nM using specific buffers and reagents (C10511-05, RIBOBIO, Guangzhou, China), and the corresponding experimental intervention was carried out. The transfection efficiency was verified by real-time quantitative polymerase chain reaction (RT-qPCR) and Western blot, including knockdown or overexpression effects.

### RT–qPCR

Total RNA from frozen kidney tissues and cell lines was extracted *via* total RNA isolation reagent (15596018CN, Invitrogen, CA, USA). For mRNA, 1,000 ng of total RNA was reverse transcribed *via* HIScript III RT SuperMix (R323, Vazyme, Nanjing, China). RT–qPCR for target genes was subsequently performed *via* ChamQ Universal SYBR qPCR Master Mix (Q711, Vazyme, Nanjing, China), and the expression levels were subsequently normalized to that of β-Actin. Specific primers were designed to distinguish STAT3 pre-mRNA (spanning an exon-intron junction) and mature mRNA (spanning an exon-exon junction). RNA samples were treated with RNase-free DNase I (D7070, Beyotime, Shanghai, China) to eliminate genomic DNA contamination prior to reverse transcription. All the mRNA primers used were designed, constructed, and validated by GeneRay Biotech Co., Ltd. (Shanghai, China), and the sequences are listed in supplementary Table S1.

### Western blot

The protein suspensions were lysed, centrifuged, and collected, and the protein concentrations of all the samples were determined *via* a bicinchoninic acid protein assay kit (KTD3001, Abbkine, Wuhan, China). Depending on the molecular weight of the target protein, an 8% or 12% gel was made, loaded with 30 µg of total protein per well, separated by electrophoresis and subsequently transferred to a polyvinylidene fluoride membrane (IPVH00010, Millipore, Boston, USA). After blocking, antibody incubation, and washing, the bands were exposed under a gel imager. Immunoblot analysis was performed with the following primary antibodies: anti-FN (1:1000, ab2413, Abcam, Cambridge, UK), anti-Col I (1:750, sc-59772, Santa Cruz, Dallas, USA), anti-E-cadherin (1:1000, AF0131, Affinity, Changzhou, China), anti-vimentin (1:1000, BF8006, Affinity, Changzhou, China), anti-alpha-smooth muscle actin (α-SMA) (1:1000, AF1032, Affinity, Changzhou, China), anti-GPX4 (1:1000, ab125066, Abcam, Cambridge, UK), anti-SLC7A11 (1:1000, DF12509, Affinity, Changzhou, China), anti-ACSL4 (1:750, sc-365230, Santa Cruz, Dallas, USA), and anti-STAT3 (1:750, sc-8019, Santa Cruz, Dallas, USA). The secondary antibodies used in the experiments were horseradish peroxidase-conjugated goat anti-mouse (1:15000, A21010, Abbkine, Wuhan, China) and goat anti-rabbit (1:15000, A21020, Abbkine, Wuhan, China) antibodies. β-Tubulin (1:750, AF2839, Beyotime, Shanghai, China) served as an internal control. The protein bands were quantitatively analyzed using Image J 1.53t (https://imagej.nih.gov/ij).

### GSH assay

The cellular redox status was assessed *via* a GSH content assay kit (KTB1600, Abbkine, Wuhan, China). The cell suspension was lysed in precooled extraction buffer, followed by centrifugation and collection of the upper clarified liquid layer. Standards were prepared at a concentration of 200 µg/mL as described in the kit instructions. The samples were added sequentially to a 96-well plate, standard wells were set up in a concentration gradient, and a standard curve was plotted on the basis of the absorbance values of each well. The absorbance values of the remaining blank and sample wells were also measured at 412 nm, and the GSH content of each sample was calculated according to the previously plotted standard curve.

### Malondialdehyde (MDA) assay

A micro lipid peroxidation assay kit (KTB1050, Abbkine, Wuhan, China) was used to assess the level of lipid peroxidation in the cells. A total of 1 × 10^7^ cells were collected, and the cells were washed with phosphate-buffered saline and centrifuged to discard the supernatant. The lower cell precipitate was retained, and 0.5 mL of precooled extraction buffer was added. The supernatant was collected, and a working solution was prepared for further analysis. All the samples were incubated in a water bath at 95 °C for 30 min, followed by another 10 min of centrifugation. For each sample, 200 μL of supernatant was pipetted and added sequentially to a 96-well plate, and the absorbance values of all the wells were measured at 532 nm and 600 nm to calculate the MDA content of all the samples according to the formula.

### Ferrous ion (Fe^2+^) assay

An Fe^2+^ content assay kit (KTB1116, Abbkine, Wuhan, China) was used to assess intracellular iron accumulation. Approximately 1 × 10^7^ cells were collected, and the supernatant was obtained by discarding the lower cell precipitate after sufficient washing and centrifugation in phosphate-buffered saline, adding 0.5 mL of extraction buffer, placing on ice for lysis for at least 10 min, and mixing with brief shaking during the period. Standard wells were prepared, samples were added sequentially, the absorbance values of all the wells were measured at 593 nm, the standard curve was plotted with the concentration of standard solution and absorbance values of the standard wells, and the calculation of the cellular Fe^2+^ content was completed according to the formula.

### Reactive oxygen species (ROS) assay

After setting up the blank control, the optimal configuration concentration of the cell ROS probe was determined. According to the manufacturer’s instructions (S0033S, Beyotime, Shanghai, China), the probe was applied to RTECs and fully incubated. The cellular ROS levels were evaluated using a flow cytometer.

### Transmission electron microscopy (TEM)

Digest, collect the cells, and add 2.5% glutaraldehyde solution. Further fix them, then centrifuge, dehydrate, and prepare sections for staining (G1102, Servicebio, Wuhan, China). The sample detection work was completed by the Electron Microscopy Core Laboratory of Nanjing Medical University.

### Obtaining potential targets of bufalin and DKD

The chemical structure and canonical SMILES of bufalin were downloaded from PubChem (https://pubchem.ncbi.nlm.nih.gov/). These information were then imported into the SwissTargetPrediction (http://www.swisstargetprediction.ch/) to identify the targets related to bufalin. The platform uses 2D and 3D molecular similarity methods to calculate the probability of interaction with known ligands. We adopted the default probability threshold of this platform and finally retained all the predicted targets for subsequent analysis to ensure the breadth of the analysis. The potential targets of DKD were obtained from the Genetic Association Database (https://www.genecards.org/). The potential core target set of bufalin in DKD was obtained by intersecting the bufalin target dataset with the DKD disease target dataset. This core target set was then submitted to the Metascape online platform for Gene Ontology (GO) and Kyoto Encyclopedia of Genes and Genomes (KEGG) pathway enrichment analyses. To evaluate the association with ferroptosis, the core target set was cross-referenced with the known ferroptosis regulator gene sets from the FerrDb V2 database (http://www.zhounan.org/ferrdb/), identifying a ferroptosis-related gene subset within the core targets along with their regulatory types [26].

### Molecular docking method

The binding mode and binding force of bufalin and STAT3 were investigated through computer simulation. Firstly, the protein structure of STAT3 was downloaded from Protein Data Bank (https://www.rcsb.org/), and the structure data file of bufalin was downloaded from PubChem (https://pubchem.ncbi.nlm.nih.gov/). The binding situation between bufalin and STAT3 was predicted by using CB-Dock2 (https://cadd.labshare.cn/cb-dock2/php/ index.php) through the blind docking principle [27].

### Dual-luciferase reporter assay

Based on the identification of STAT3 as a downstream target of bufalin from preliminary analyses, the full-length human STAT3 3′ untranslated region (UTR) (transcript: NM_139276.3) was amplified by PCR. This fragment was cloned into the multiple cloning site downstream of the luciferase gene in a promoterless reporter vector to generate the wild-type construct (pGL3-STAT3-3′ UTR-WT). To serve as a rigorous negative control, a scrambled version was designed by completely randomizing the native nucleotide sequence while preserving its length and base composition using bioinformatics algorithms. This scrambled sequence was synthesized and inserted into the identical vector backbone using the same cloning strategy, yielding the control construct (pGL3-STAT3-3′ UTR-SCR). The 293 T cells were cultured in a 96-well plate and transfected using Lipo8000 (C0533, Beyotime, Shanghai, China). After processing the cells and collecting and lysing them, the relative luciferase activity was detected using the dual-luciferase reporter assay kit (RG027, Beyotime, Shanghai, China).

### Measurement of STAT3 mRNA stability

To determine the half-life of STAT3 mRNA, an actinomycin D (Act D) chase assay was performed. Briefly, cells were pretreated with DMSO or bufalin under HG conditions, followed by exposed to a concentration of 5 μg/mL Act D (A1410, Sigma-Aldrich, St. Louis, USA) to block *de novo* RNA synthesis. Total RNA was isolated at sequential time points (0, 2, 4, 8, 16, and 24 h) post-Act D addition using TRIzol reagent. STAT3 mRNA levels at each time point were quantified by RT-qPCR, normalized to the stable reference gene β-Actin. The decay rate constant (k) was derived by fitting the time-course mRNA data to a single-phase exponential decay model using nonlinear regression (version 8.0, GraphPad Software, San Diego, USA), and the half-life was calculated.

### Statistical analysis

Adobe Illustrator 2022, Figdraw, and GraphPad Prism 8.0 were used for data analysis and image editing. All quantitative data in this work were ultimately expressed as the mean ± standard deviation and individual data points were clearly marked in the charts to show the data distribution. Comparisons between two groups were made *via* the unpaired Student’s *t* test, and comparisons between multiple groups were made *via* one-way analysis of variance. Sidak’s multiple comparison test was further used for pairwise comparisons. *p* < 0.05 indicated that the difference was statistically significant.

## Results

### Bufalin ameliorated renal dysfunction and attenuated the progression of TIF

**Figure 1.**
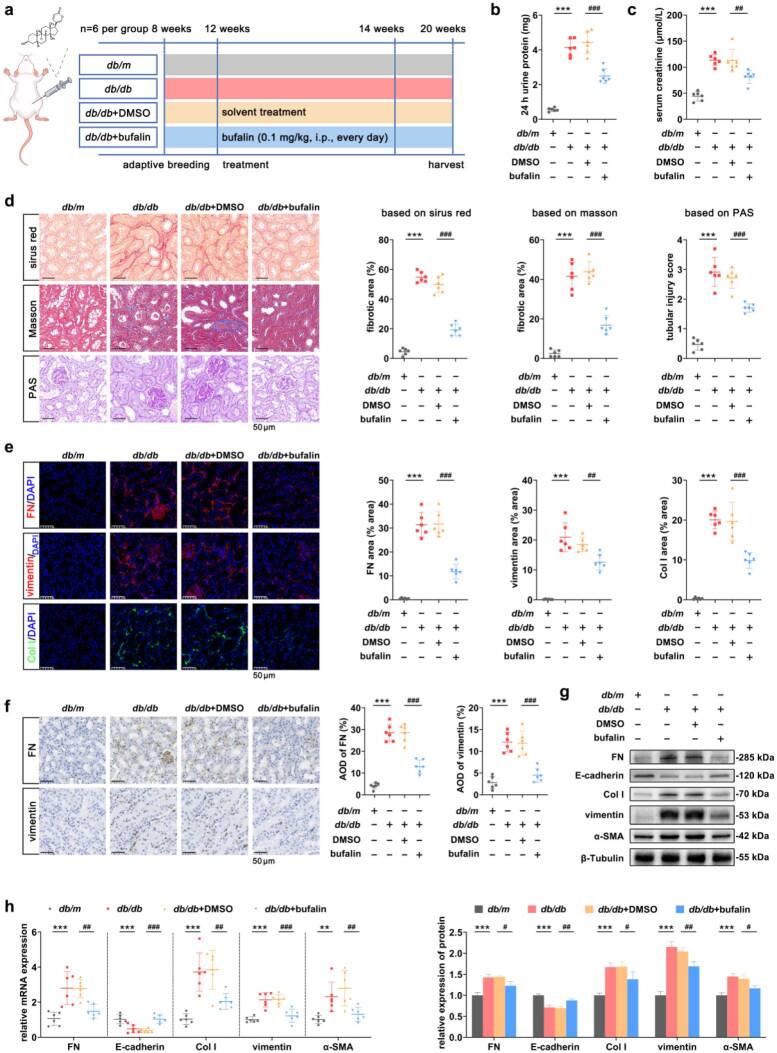
Bufalin ameliorated renal dysfunction and attenuated the progression of TIF. (a) Schematic overview of the experiment design of the DKD mice model. (b and c) 24-h urine protein and SCr levels in each group of mice. (d) Representative images of pathologic staining show that bufalin ameliorates TIF, including reduction of ECM deposition. Scale bar, 50 μm. The scores for the tubular damage and fibrotic area were calculated. (e) Representative images of IF of FN, vimentin, and Col I in the kidney tissues of mice. Scale bar, 50 μm. The fluorescence density of the TIF-related indicators was quantified. (f) IHC of FN and vimentin with corresponding quantifications. (g) The protein expression levels of TIF-related indicators in the kidney tissues of mice were measured by Western blot. (h) The relative mRNA expression levels of TIF-related indicators were measured by RT-qPCR. For *in vivo* experiments, *n* = 6 per group; for cellular experiments, *n* = 4. The data are expressed as the mean ± SD. **p < 0.01, ***p < 0.001, ^#^*p* < 0.05, ^##^*p* < 0.01, ^###^*p* < 0.001 vs. the indicated group.

In this study, after determining the administration method of bufalin, we verified its effect based on the successfully established DKD model (Figure 1(a)). *db/db* mice showed significantly higher levels of 24-h urine protein and SCr compared with those in the *db/m* group, and the urine protein and SCr levels of mice in the *db/db*+bufalin group were significantly lower compared with those in the *db/db*+DMSO group (Figure 1(b) and (c)). Under the light microscope, the histopathological morphology of the kidneys of mice in the *db/db* group changed, with increased collagen deposition and fibrosis in the tubular interstitium of the kidneys, while the histopathological changes of the kidneys of mice in the *db/db*+bufalin group were attenuated (Figure 1(d)). In addition, the fluorescence intensity of FN, vimentin, and Col I was significantly enhanced compared with that of the *db/m* group, while the fluorescence intensity of the *db/db*+bufalin group was significantly reduced compared with that of the *db/db*+DMSO group (Figure 1(e)). The results of IHC showed that there was a consistent trend of change in the expression of FN and vimentin as compared with those of IF (Figure 1(f)). In terms of mRNA and protein expression, the expression levels of FN, Col I, a-SMA, and vimentin were significantly higher in the *db/db* group compared to the *db/m* group, whereas the levels of all indicators were significantly altered in the *db/db*+bufalin group compared to the *db/db*+DMSO group (Figure 1(g) and (h)).

### Effect of bufalin on RTECs cellular viability and HG-induced TIF

**Figure 2.**
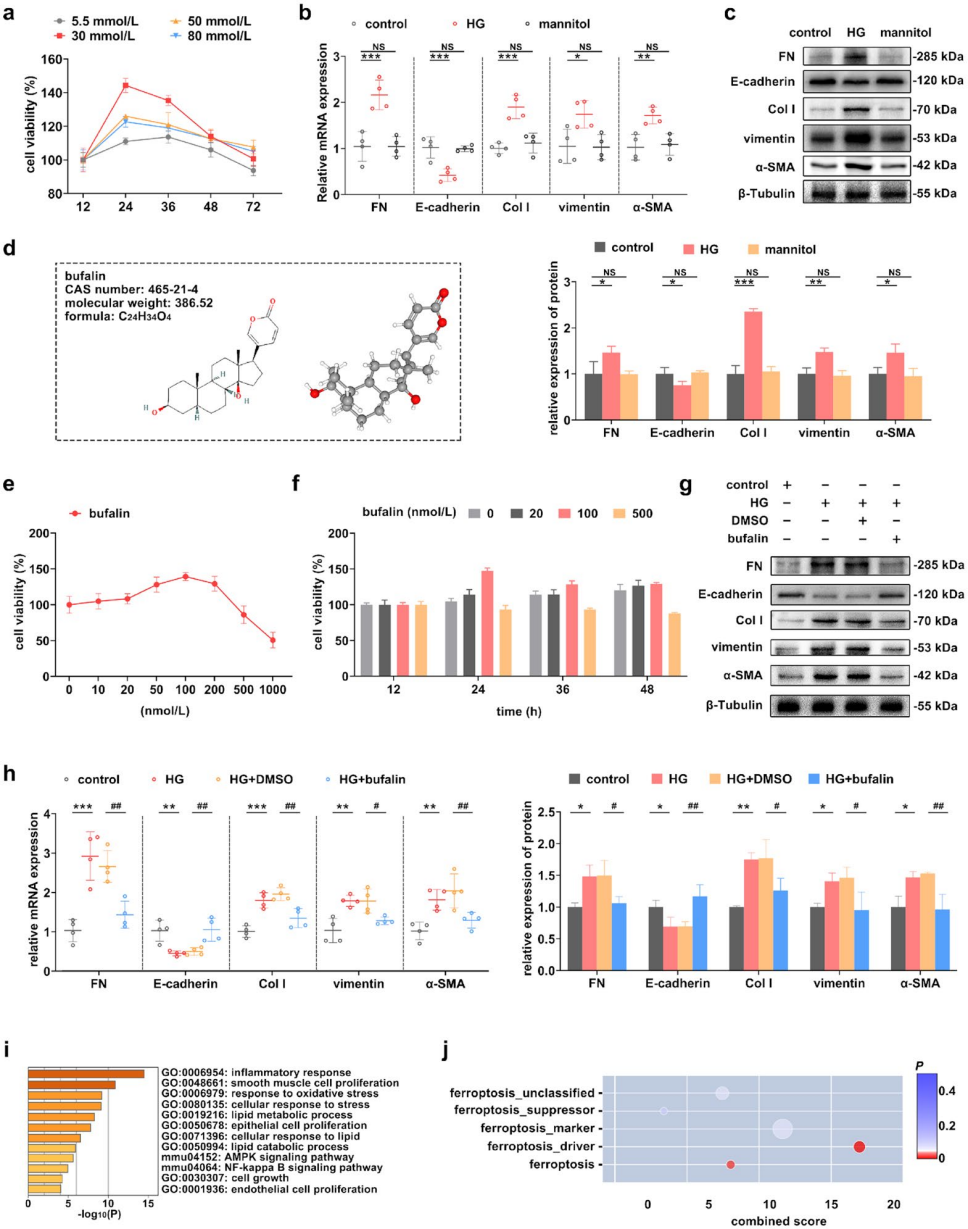
Effect of bufalin on RTECs cellular viability and HG-induced TIF. (a) Cell viability of RTECs under different conditions. (b) The mRNA expression levels of TIF-related indicators were assessed by RT–qPCR in HG-induced RTECs. (c) The TIF-related indicators were assessed by Western blot in HG-induced RTECs, and the protein levels were detected. (d) Molecular structure and information of bufalin. (e and f) The viability of RTECs treated with different bufalin doses (0, 10, 20, 50, 100, 200, 500, and 1,000 nmol/L) after different treatment time (12, 24, 36, and 48 h) was detected by CCK-8. (g and h) The expression levels of TIF-related indicators after the treatment of bufalin in HG-induced RTECs, and the protein levels were detected. (i) Functional analysis of downstream targets of bufalin. (j) Correlation analysis of downstream targets of bufalin and ferroptosis. For *in vivo* experiments, *n* = 6 per group; for cellular experiments, *n* = 4. The data are expressed as the mean ± SD. *p < 0.05, **p < 0.01, ***p < 0.001, ^#^*p* < 0.05, ^##^*p* < 0.01 vs. the indicated group. NS, non-significant.

RTECs cultured under the conditions of HG showed significant proliferation. The results obtained through CCK-8 assay indicated that a significant increase in cell viability was observed when the glucose concentration was 30 mmol/L and the intervention lasted for 24 h (Figure 2(a)). Under these conditions, an *in vitro* model of DKD was established. The results of RT–qPCR and Western blot revealed that the expression levels of FN, Col I, vimentin, and a-SMA were significantly increased, while the expression of E-cadherin was significantly decreased in the cells of the HG group compared to the control group, and none of the expression levels in the mannitol group were significantly changed, ruling out the effect of high osmolarity on the cells (Figure 2(b) and (C)). The information of bufalin was obtained from PubChem (Figure 2(d)). To evaluate the cytotoxic effect of bufalin on RTECs, different concentrations of bufalin (with the highest concentration being 1,000 nmol/L) were used, and the cells were continuously monitored for 48 h. The CCK-8 results indicated that at concentrations lower than 100 nmol/L (Figure 2(e)), bufalin had no significant cytotoxicity on RTECs, and it showed a gradually increasing trend over time (Figure 2(f)). After using bufalin in the HG-treated RTECs, compared with the HG+DMSO group, the expression levels of FN, Col I, vimentin, and α-SMA in the cells of the HG+bufalin group were significantly decreased, while the expression of E-cadherin was significantly increased (Figure 2(g) and (h)). Through network pharmacology analysis, the potential target genes of bufalin were identified. Further analysis revealed that key signaling pathways, including PTGS2, STAT, and mTOR, are implicated in the regulation of oxidative stress and lipid metabolism. The functional analysis and correlation analysis of candidate genes suggested that the effect of bufalin on TIF is highly likely to involve the regulation of ferroptosis (Figure 2(i), (j) and supplementary Table S2).

### Inhibition of ferroptosis in RTECs reversed the alterations in markers associated with TIF

**Figure 3.**
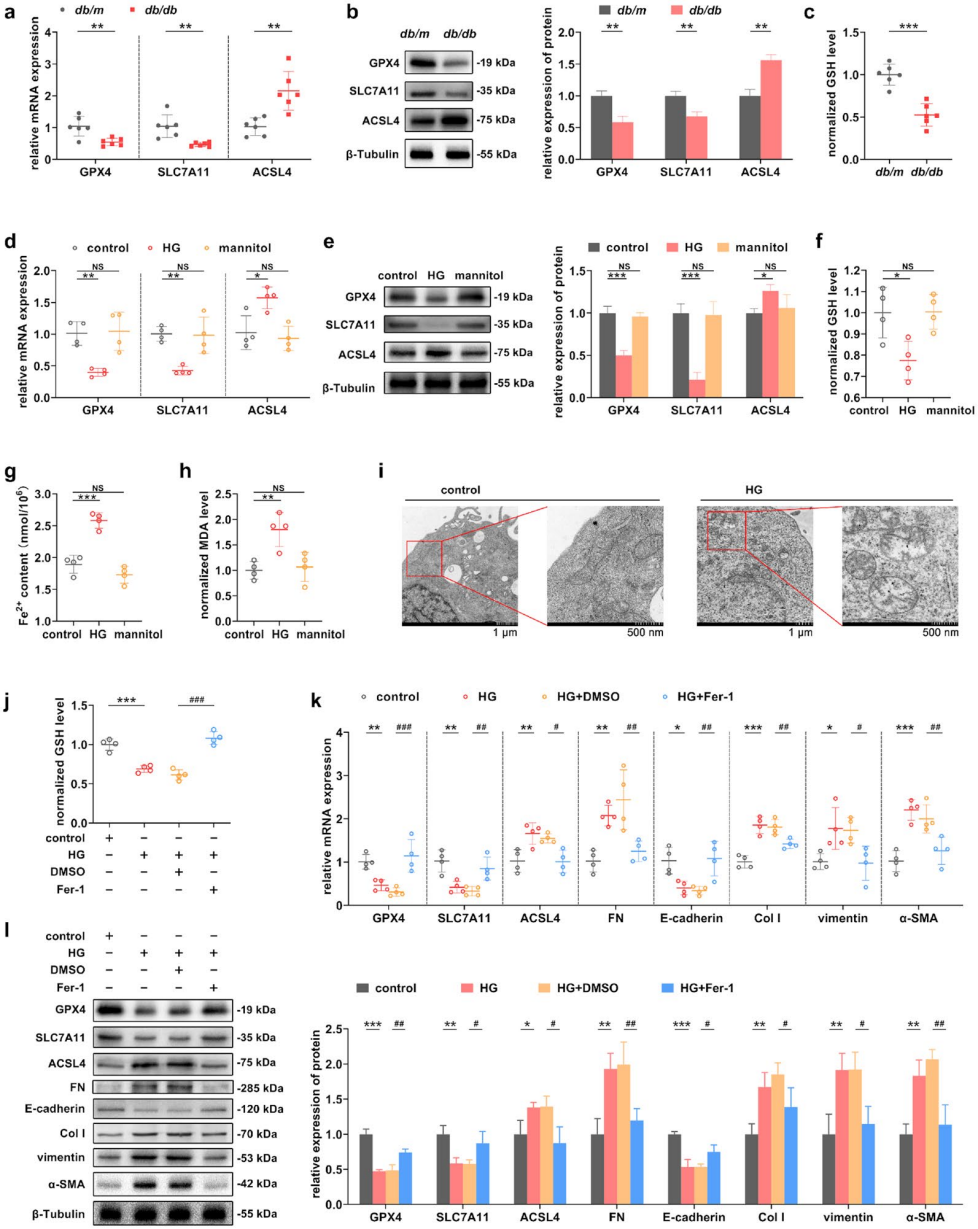
Inhibition of ferroptosis in RTECs reversed the alterations in markers associated with TIF. (a and b) RT–qPCR and Western blot were used to measure the expression levels of ferroptosis-related indicators in the kidney tissues of *db/db* mice. (c) GSH levels in mice. (d and e) The expression levels of ferroptosis-related indicators were analyzed by RT–qPCR and Western blot in HG-induced RTECs. (f, g, and h) The contents of GSH, Fe^2+^ and MDA were measured in HG-induced RTECs. (i) TEM revealed significant changes in mitochondrial morphology in HG-induced RTECs. The scale bars were located at the bottom of the image. (j) Changes in cellular GSH levels after the use of Fer-1 in HG-induced RTECs. (k and l) The mRNA and protein expression levels of ferroptosis-related indicators in HG-induced RTECs were determined by RT-qPCR and Western blot to verify the role of Fer-1, and further analysis of TIF-related indicators was used to assess the effect of ferroptosis on TIF. For *in vivo* experiments, *n* = 6 per group; for cellular experiments, *n* = 4. The data are expressed as the mean ± SD. *p < 0.05, **p < 0.01, ***p < 0.001, ^#^*p* < 0.05, ^##^*p* < 0.01, ^###^*p* < 0.001 vs. the indicated group. NS, non-significant.

The occurrence of ferroptosis was first assessed, and the results showed that the expression level of ACSL4 was significantly higher in the *db/db* group compared with the *db/m* group, while the expression of GPX-4 and SLC7A11 was significantly lower (Figure 3(a) and (b)). A synchronized decrease in GSH levels occurred in *db/db* mice (Figure 3(c)). *In vitro*, the expression of ACSL4 was significantly higher in the HG group, while the expression of GPX-4 and SLC7A11 was significantly lower (Figure 3(d) and (e)). In addition, the cellular GSH contents were significantly reduced, and the contents of Fe^2+^ and MDA were significantly elevated in the HG group (Figure 3(f)–(h)). Furthermore, under TEM, the intracellular morphology, including mitochondria, was approximately normal in the control group, whereas the mitochondrial morphology was altered in the HG group, with smaller and rounded mitochondria, increased membrane density, and broken mitochondrial cristae that had nearly disappear (Figure 3(i)). After the intervention of Fer-1, the GSH levels of the HG+Fer-1 group were significantly higher than that of the HG+DMSO group (Figure 3(j)). The results of RT–qPCR and Western blot showed that the expression levels of the ferroptosis-related indicators changed significantly in the HG+Fer-1 group compared with that of the HG+DMSO group (Figure 3(k) and (l)). On this basis, the mRNA and protein expression levels of FN, Col I, vimentin, and a-SMA in the HG+Fer-1 group were significantly reduced, while the expression of E-cadherin was significantly increased (Figure 3(k) and (l)).

### Inhibition of ferroptosis ameliorated renal dysfunction and alleviated TIF *in vivo*

**Figure 4.**
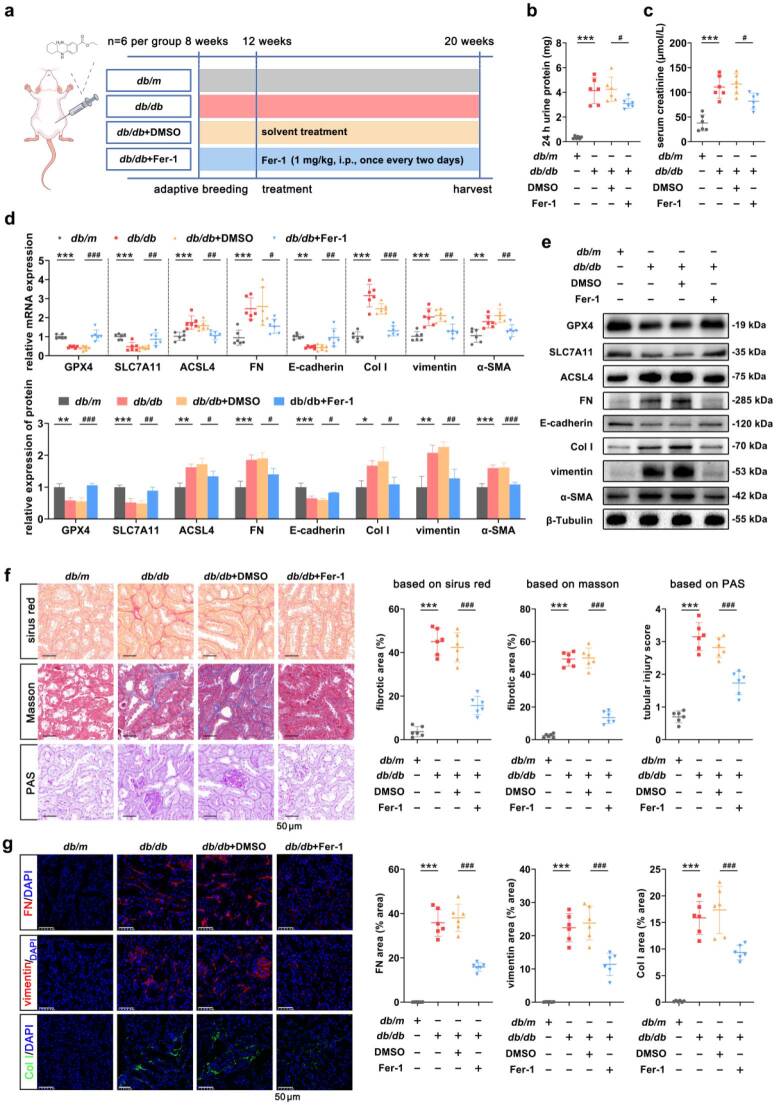
Inhibition of ferroptosis ameliorated renal dysfunction and alleviated TIF *in vivo*. (a) The schematic of the Fer-1 experimental design. (b and c) Changes in renal function in mice were determined after the application of Fer-1. (d and e) The expression levels of ferroptosis and TIF-related indicators were measured by RT–qPCR and Western blot in mice to verify the role of Fer-1. (f) Representative images of pathologic staining showing the role of Fer-1 in TIF. Scale bar, 50 μm. The tubular damage score and fibrotic area were quantified. (g) IF of FN, vimentin, and Col I in renal cotex. Scale bar, 50 μm. The fluorescence intensity was quantified. For *in vivo* experiments, *n* = 6 per group. The data are expressed as the mean ± SD. *p < 0.05, **p < 0.01, ***p < 0.001, ^#^*p* < 0.05, ^##^*p* < 0.01, ^###^*p* < 0.001 vs. the indicated group.

The effects of ferroptosis on renal function and TIF in *db/db* mice were observed after the intraperitoneal injection of Fer-1 (Figure 4(a)). In terms of renal function, 24-h urine protein and SCr levels were significantly lower in the *db/db*+Fer-1 group of mice compared to the *db/db*+DMSO group (Figure 4(b) and (c)). With respect to the inhibitory effect of Fer-1 on ferroptosis, the results showed that the expression of ACSL4 was significantly lower and that the expression of GPX-4 and SLC7A11 was significantly increased in the *db/db*+Fer-1 group than in the *db/db*+DMSO group (Figure 4(d) and (e)). Here, TIF-related indicators were detected synchronously. Compared with those in the *db/db*+DMSO group, the expression levels of FN, Col I, vimentin, and a-SMA were significantly lower and the expression of E-cadherin was significantly increased in the *db/db*+Fer-1 group (Figure 4(d) and (e)). In terms of histopathology, mice in the *db/db*+Fer-1 group presented reduced levels of TIF and decreased interstitial collagen deposition (Figure 4(f)). Further IF revealed that the expression levels of FN, vimentin and Col I were significantly reduced in the *db/db*+Fer-1 group compared with the *db/db*+DMSO group (Figure 4(g)). *In vivo* experiments have reaffirmed that ferroptosis serves as a crucial determinant in the progression of TIF.

### Inhibition of ferroptosis is central to bufalin-mediated protection against TIF

**Figure 5.**
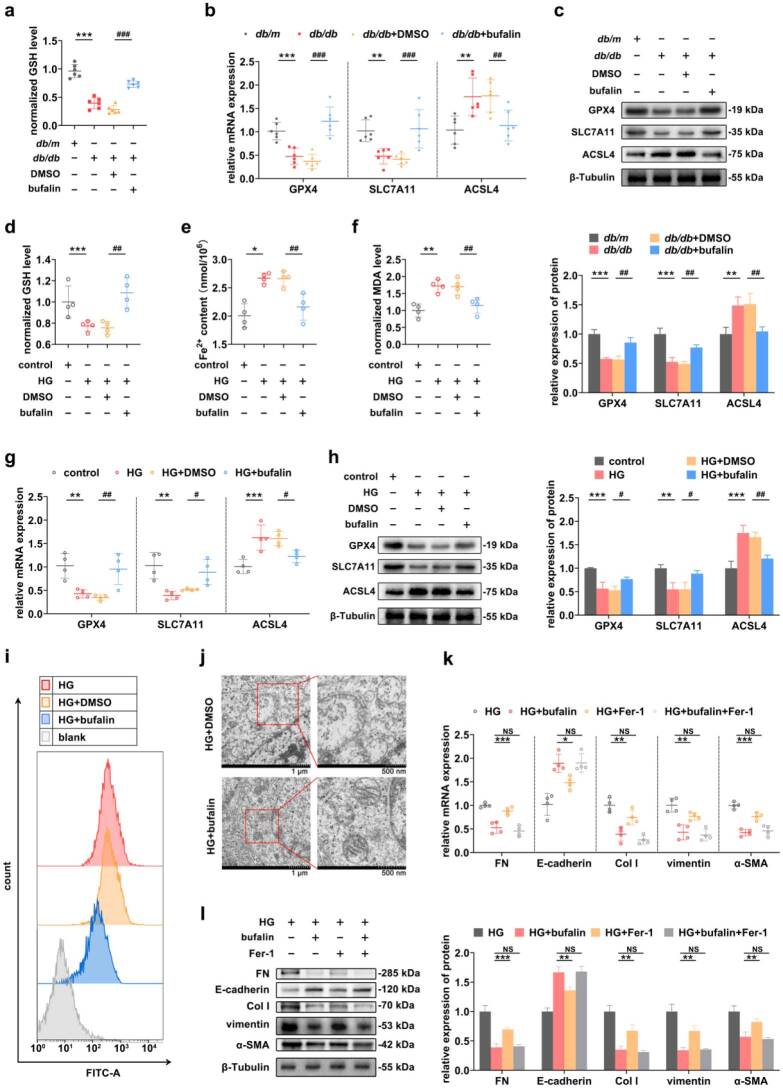
Inhibition of ferroptosis is central to bufalin-mediated protection against TIF. (a) GSH levels in *db/db* mice. (b and c) RT–qPCR and Western blot analysis of ferroptosis-related indicators were performed to evaluate the role of bufalin in ferroptosis in DKD mice. (d, e, and f) The cellular contents of GSH, Fe^2+^ and MDA verified the role of bufalin in ferroptosis. (g and h) The inhibitory effect of bufalin on ferroptosis in HG-treated RTECs was demonstrated by RT–qPCR and Western blot. (i) ROS levels in bufalin-treated RTECs. (j) TEM revealed mitigated mitochondrial changes after the intervention of bufalin. The scale bar is located at the bottom of the image. (k and l) The expression changes of TIF-related indicators after co-treatment with bufalin and Fer-1 were detected by RT-qPCR and Western blot. For *in vivo* experiments, *n* = 6 per group; for cellular experiments, *n* = 4. The data are expressed as the mean ± SD. *p < 0.05, **p < 0.01, ***p < 0.001, ^#^*p* < 0.05, ^##^*p* < 0.01, ^###^*p* < 0.001 vs. the indicated group. NS, non-significant.

The role of bufalin in the development of ferroptosis in *db/db* mice was therefore evaluated. The results showed that the contents of GSH were higher in the *db/db*+bufalin group than in the *db/db*+DMSO group (Figure 5(a)). The mRNA and protein expression of ACSL4 was significantly lower in the *db/db*+bufalin group, and the expression of GPX4 and SLC7A11 was significantly higher (Figure 5(b) and (c)). *In vitro*, the results showed that the cellular GSH levels were significantly higher and the contents of Fe^2+^ and MDA were significantly lower in the HG+bufalin group compared to the HG+DMSO group (Figure 5(d)–(f)). In addition, the expression levels of ferroptosis-related indicators were significantly altered in the HG+bufalin group compared with the HG+DMSO group (Figure 5(g) and (h)). Flow cytometry analysis also indicated that under the intervention of bufalin, the level of ROS decreased (Figure 5(i)). Under the TEM, the mitochondrial morphology in the HG+DMSO group showed increased membrane density and decreased cristae, whereas the mitochondrial ridges in the cells of the HG+bufalin group were clearly visible, and the morphological changes such as atrophy and increased membrane density were alleviated (Figure 5(j)), further confirming the inhibitory effect of bufalin on ferroptosis in DKD models. The co-treatment results of bufalin and Fer-1 showed that the mRNA and protein expression levels of TIF-related indicators in the HG+bufalin + Fer-1 group did not change significantly, and no stronger protective effect was observed compared to the bufalin-only group (Figure 5(k) and (l)). Functional rescue experiments demonstrated that inhibition of ferroptosis, while essential, is not the sole mechanism through which bufalin alleviates TIF.

### Knockdown of the target gene STAT3 inhibited ferroptosis and TIF induced by HG

**Figure 6.**
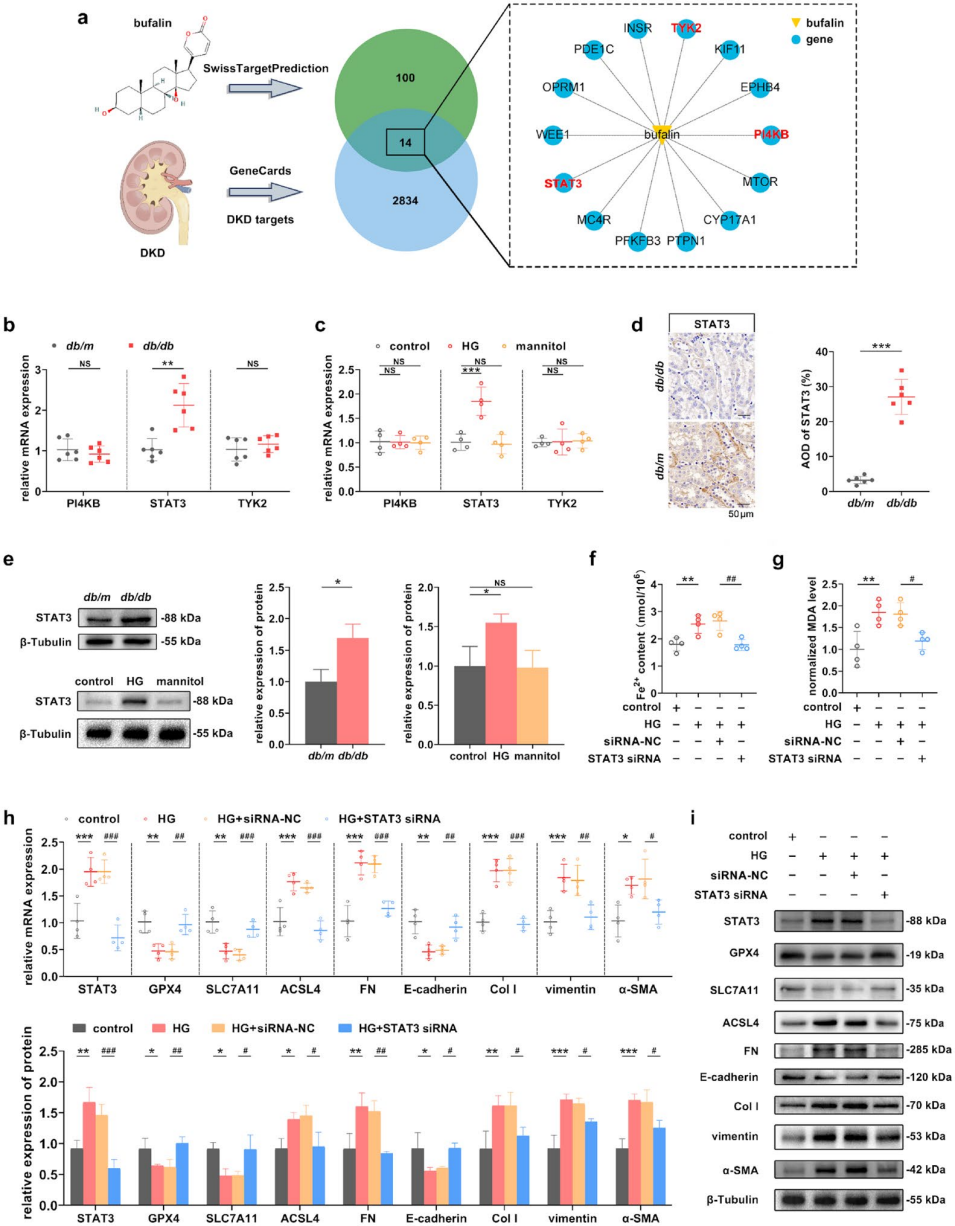
Knockdown of the target gene STAT3 inhibited ferroptosis and TIF induced by HG. (a) Through network pharmacology analysis and GeneCards screening, the potential targets regulated by bufalin on TIF were identified, and a total of 14 targets were shown. (b and c) The expression levels of the top three targets were detected in DKD models by RT-qPCR. (d) IHC of STAT3 in renal cotex, and scale bar, 50 μm. (e) The mRNA and protein expression levels of STAT3 in DKD models. (f and g) The detection of Fe^2+^ and MDA contents in cells was used to assess the occurrence of ferroptosis after the knockdown of STAT3. (h and i) The mRNA and protein expression levels of ferroptosis and TIF-related indicators after the knockdown of STAT3 were detected by RT-qPCR and Western blot. For *in vivo* experiments, *n* = 6 per group; for cellular experiments, *n* = 4. The data are expressed as the mean ± SD. *p < 0.05, **p < 0.01, ***p < 0.001, ^#^*p* < 0.05, ^##^*p* < 0.01, ^###^*p* < 0.001 vs. the indicated group. NS, non-significant.

The results of network pharmacology analysis showed that bufalin acted on 100 target genes, among which 14 targets were highly correlated with the occurrence and development of DKD. The top 3 target genes with relatively higher scores were PIK4B, STAT3, and TYK2 (Figure 6(a) and supplementary table S3). The RT-qPCR, Western blot, and IHC results indicated that the expression levels of STAT3 in *db/db* mice and HG-induced RTECs were significantly increased (Figure 6(b)–(e)). To investigate the role of STAT3 in ferroptosis and TIF in DKD models, STAT3 siRNA was employed to knock down the expression of STAT3 in HG-induced RTECs. After the knockdown of STAT3, compared with the HG+siRNA NC group, the contents of Fe^2+^ and MDA in the cells were significantly decreased (Figure 6(f) and (g)), and the expression levels of proteins and mRNAs related to ferroptosis in the HG+STAT3 siRNA group were significantly changed (Figure 6(h) and (i)). Here, we simultaneously verified the association between STAT3 and TIF. Compared with the HG+siRNA NC group, the expression levels of EMT and ECM-related indicators in the HG+STAT3 siRNA group were significantly changed. The knockdown efficiency has been verified through RT-qPCR and WB (Figure 6(h) and (i)).

### Bufalin downregulates STAT3 *via* post-transcriptional regulation

**Figure 7.**
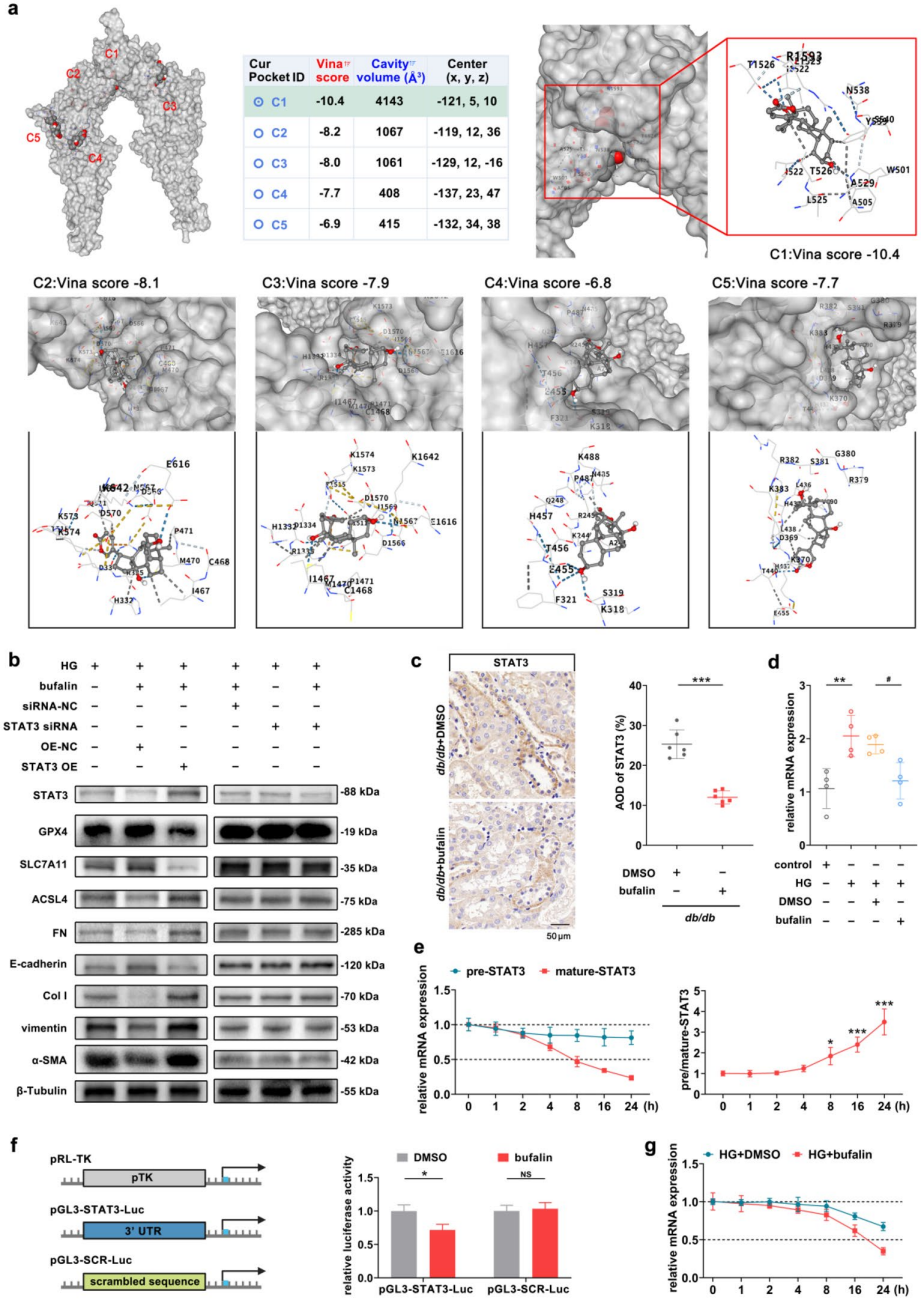
Bufalin downregulates STAT3 *via* post-transcriptional regulation. (a) The binding sites of bufalin and STAT3 that could be interacted with were predicted by molecular docking method. The binding sites, conformations, and docking scores are presented here. (b) In RTECs pretreated with bufalin, STAT3 overexpression plasmid or STAT3 siRNA were respectively transfected. The protein expression levels of ferroptosis and TIF-related indicators were detected by Western blot. (c) The expression changes of STAT3 in the kidney tissues of mice treated with bufalin were detected by IHC. Scale bar, 50 μm. (d) RT-qPCR was used to detect the mRNA expression level of STAT3 under the intervention of bufalin. (e) Detection and ratio analysis of STAT3 precursor mRNA and mature mRNA expression. (f) The binding of bufalin and STAT3 3′ UTR was verified by dual-luciferase reporter assay. (g) The mRNA stability of STAT3 in RTECs treated with bufalin was significantly decreased. For *in vivo* experiments, *n* = 6 per group; for cellular experiments, *n* = 4. The data are expressed as the mean ± SD. *p < 0.05, **p < 0.01, ***p < 0.001, ^#^*p* < 0.05 vs. the indicated group. NS, non-significant.

Molecular docking successfully predicted the binding conformation of bufalin with STAT3 and identified five distinct interaction sites (Figure 7(a)). To functionally validate STAT3 as the key downstream mediator, we generated a STAT3 overexpression (OE) plasmid and co-treated HG-exposed RTECs with bufalin and the STAT3 OE plasmid. Compared with the HG+bufalin + STAT3 OE-NC group, the indicators related to ferroptosis and TIF showed significant reversals, and even exhibited worse expression than the HG group (Figure 7(b) and Supplementary Fig. S1). Importantly, complementary loss-of-function experiments—co-transfection of bufalin with STAT3 siRNA—recapitulated the same phenotypic effects on ferroptosis and TIF (Figure 7(b) and Supplementary Fig. S1). IHC analysis demonstrated a marked reduction in STAT3 protein expression in the *db/db*+bufalin group relative to the *db/db*+DMSO group (Figure 7(c)). Consistently, RT-qPCR revealed that bufalin treatment significantly suppressed STAT3 mRNA levels in HG-treated RTECs compared with the HG+DMSO group (Figure 7(d)). To elucidate the underlying regulatory mechanism, kinetic analyses of nascent and mature STAT3 transcripts showed that bufalin did not alter nascent pre-mRNA levels but induced a rapid and substantial decrease in mature STAT3 mRNA, resulting in a significantly increased pre-mRNA/mature mRNA ratio (Figure 7(e)). Critically, dual-luciferase reporter assays indicated that bufalin specifically reduced luciferase activity driven by the wild-type STAT3 3′ UTR, whereas no significant effect was observed with the scrambled control construct (Figure 7(f)). This clear dissociation between unchanged transcriptional output (nascent RNA) and diminished steady-state mRNA abundance definitively rules out transcriptional repression as the primary mechanism and strongly supports post-transcriptional regulation *via* mRNA destabilization. Act D chase experiments further confirmed that bufalin markedly accelerated STAT3 mRNA decay: The half-life of STAT3 mRNA decreased from 44.26 h (95% CI: 36.45–55.81) in the HG+DMSO group to 18.93 h (95% CI: 16.00–22.80) in the HG+bufalin group—a statistically significant shortening (Figure 7(g)). Collectively, these gain- and loss-of-function approaches provide convergent genetic evidence that bufalin inhibits ferroptosis and alleviates TIF predominantly through STAT3-dependent post-transcriptional regulation.

## Discussion

**Figure 8.**
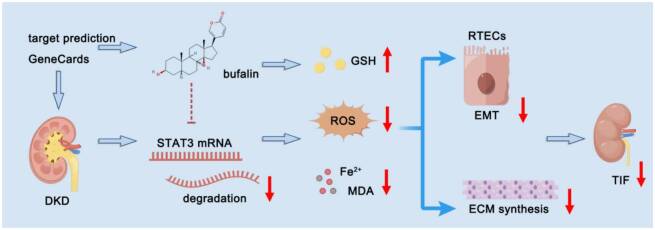
Schematic diagram illustration of the mechanism by which bufalin regulates STAT3-mediated ferroptosis in DKD.

Determining the pathogenesis of DKD and developing new targeted therapies to inhibit EMT and reduce excessive deposition of ECM are important for improving the prognosis of patients [1]. Bufalin is widely used in clinical practice as a component of traditional Chinese medicine, and in recent years, it has been increasingly reported in tumors; however, it has been less studied in mitigating intrinsic renal cell injury but still has the potential to play a nephroprotective role. In a model of adriamycin-induced nephropathy, bufalin may reduce urinary protein excretion by protecting the glomerular filtration barrier and glomerular filtration function [28]. In addition, bufalin can inhibit the proliferation of mesangial cells by regulating the progression of the cell cycle, suggesting that bufalin may be a potential therapeutic agent for the prevention of mesangial proliferative glomerulonephritis [10]. Given that bufalin is a Na^+^/K^+^-ATPase inhibitor and that aberrant activation of tubular Na^+^/K^+^-ATPase is a pathological feature in advanced DKD driven primarily by RTECs, we therefore selected this cell type as our *in vitro* model to directly investigate its mechanism of action. Here, a previously unrecognized mechanism linking bufalin to TIF in DKD has been demonstrated (Figure 8). Our study confirmed the protective effect of bufalin in the kidney. The intervention of bufalin significantly improved renal function and histopathological changes in *db/db* mice, and the significant changes in EMT and ECM synthesis-related indexes further suggested that bufalin effectively alleviated TIF in DKD.

Network pharmacology analysis revealed that bufalin is closely associated with ferroptosis and TIF in DKD. Mechanistically, bufalin promotes the degradation of STAT3 mRNA *via* a post-transcriptional mechanism. As STAT3 is a key regulator of ferroptosis in DKD, bufalin attenuates the progression of TIF by inhibiting STAT3-mediated ferroptosis.

The possible mechanism by which bufalin alleviates TIF in DKD is a key question for us to consider. The biological activity of bufalin may involve the regulation of programmed cell death. In a gentamicin-induced model of acute kidney injury, bufalin alleviated cell injury by regulating pyroptosis [23]. Ferroptosis, a novel form of programmed cell death initially induced by erastin, manifests itself in cellular biochemistry as depletion of GSH and inactivation of GPX4, ultimately leading to lethal lipid peroxidation [13]. An important member of the Xc(-) system, SLC7A11, also known as xCT, mediates GSH production and attenuates oxidative stress to some extent [14,15]. During the progression of lipid metabolism, ACSL4 is an important molecule whose activation drives the iron-containing enzyme lipoxygenase to produce lipid oxides and further promotes ferroptosis [14,16]. Fer-1 is a synthetic antioxidant and a potent, selective inhibitor of ferroptosis that prevents damage to membrane lipids by a reductive mechanism, thereby inhibiting cell death [11,29,30]. Fer-1 can be used as a probe to explore the occurrence of ferroptosis in various diseases and has potential as a drug in renal diseases [31–34]. The use of Fer-1 ameliorated HG-induced EMT in human renal proximal tubular cells and rescued renal pathologic injury in DKD mice [33]. In addition, Fer-1 attenuated oxalate-induced injury, fibrosis, and calcium oxalate stone formation in RTECs by inhibiting ferroptosis [35]. After the inhibition of ferroptosis, *db/db* mice showed significantly better renal function and improved histopathology, while the expression of TIF-related indicators was reversed.

Having clarified the role of ferroptosis, we explored the possible mechanisms by which bufalin inhibits TIF in DKD. Bufalin coexisted with the mitigating effect of Fer-1 on TIF, so is bufalin involved in the regulation of ferroptosis? Since the concept of ferroptosis was proposed, an increasing number of compounds have been shown to be involved in this process of cell death, and anything that promotes or inhibits the process of lipid oxidation has been shown to be involved to some extent in the regulation of ferroptosis in cells or diseases [12]. In tumors, bufalin is considered a small molecule of reactive oxygen species and thus has antitumor activity [9,25,36]. A recent study has shown that bufalin can induce ferroptosis in breast cancer by modulating the 2,4-dienoyl-CoA reductase-SLC7A11 axis [37]. However, bufalin exhibited significant antioxidant effects by reducing MDA levels, decreasing the consumption of superoxide dismutase and GSH, inhibiting the interaction between Kelch-like ECH associated protein 1 and nuclear factor erythroid 2-related factor 2, and increasing the expression of heme oxygenase-1 [22]. The regulation of ferroptosis by bufalin may have both positive and negative effects, and the regulatory effect of bufalin on ferroptosis in the kidneys is not clear. In this study, the appropriate dose of bufalin showed protective effects on DKD cells after a 24-h intervention, but its toxic effects did indeed increase with time and concentration in the cells cultured *in vitro*. In the bufalin-treated DKD models, changes in mitochondrial morphology, lipid oxidation, and alterations in signature genes and proteins due to the onset of ferroptosis were significantly reversed. Our study suggested that bufalin inhibits ferroptosis in DKD mice and HG-induced RTECs by decreasing the content of lipid peroxidation products, inhibiting the depletion of GSH, and increasing the expression of antioxidant enzymes. In further co-treatment experiments, no synergistic or additive effect was observed upon combined application of bufalin and Fer-1 at the concentrations used. These findings suggest that inhibition of ferroptosis represents one important mechanism underlying the anti-fibrotic action of bufalin, but is not the exclusive mechanism. The absence of enhanced efficacy upon co-treatment instead highlights the potent regulatory capacity of bufalin on the ferroptosis pathway and does not diminish the critical role of ferroptosis in bufalin’s mechanism of action.

The molecular mechanism by which bufalin regulates ferroptosis and TIF is an important issue that we need to consider. STAT3 belongs to the STAT protein family and is an important transcription factor that participates in regulating various physiological and pathological processes such as cell proliferation, differentiation, immune regulation, and inflammatory responses [38–42]. Network pharmacology and bioinformatics analysis techniques indicate that STAT3 is an excellent target for bufalin. Currently, in the related therapeutic target research, isoquercitrin alleviates DKD by inhibiting STAT3 phosphorylation and dimerization [43]. In a novel model of liver injury established by the combination of lipopolysaccharide and γ-D-glutamyl‐meso‐diaminopimelic acid, the activation of STAT3 led to enhanced ferritinophagy and intracellular iron efflux blockade. This disruption of iron homeostasis resulted in intracellular iron accumulation, increased lipid peroxidation, and ultimately triggered ferroptotic cell death [44]. The inhibition of STAT3 abrogated leucine-rich repeat-containing G protein-coupled receptor 6 knockout-induced mitochondrial dysfunction and ferroptosis in diabetic mice [45]. Here, it has been verified that the expression level of STAT3 is elevated. Further knockdown significantly inhibits the expression changes of indicators related to ferroptosis. Meanwhile, the correlation between STAT3 and TIF has been confirmed. In terms of mechanism, bufalin exerts its downstream effects predominantly by suppressing STAT3 expression through a post-transcriptional mechanism. Specifically, analysis of the precursor-to-mature mRNA ratio revealed no significant change in STAT3 pre-mRNA levels upon bufalin treatment, whereas mature STAT3 mRNA was markedly reduced, indicating that bufalin acts post-transcriptionally rather than by inhibiting transcription. Consistent with this, dual-luciferase reporter assays using constructs harboring the STAT3 3′ UTR demonstrated that bufalin significantly attenuated reporter activity, implicating the 3′ UTR in mediating mRNA destabilization. Furthermore, actinomycin D chase experiments confirmed that bufalin accelerated the decay rate of STAT3 mRNA, providing direct evidence for enhanced mRNA turnover. Crucially, genetic or pharmacological restoration of STAT3 expression abolished the anti-ferroptotic and anti-fibrotic effects of bufalin, thereby establishing a causal link between STAT3 downregulation and functional outcomes. In the present study, we demonstrated that bufalin exerts its renoprotective effects against tubulointerstitial fibrosis by suppressing STAT3-mediated ferroptosis. The functional rescue experiments using STAT3 overexpression and siRNA knockdown provided critical mechanistic insights: bufalin alone was as effective as STAT3 siRNA in reducing fibrosis and ferroptosis markers, whereas forced expression of STAT3 completely abrogated the protective effects of bufalin. These findings strongly argue that the antifibrotic action of bufalin is strictly dependent on the downregulation of STAT3, rather than acting through parallel STAT3-independent pathways.

Collectively, these findings demonstrate that bufalin promotes STAT3 mRNA degradation in a 3′ UTR-dependent manner, leading to reduced STAT3 levels, inhibition of ferroptosis, attenuation of ECM accumulation and EMT in RTECs, and ultimately amelioration of TIF. This post‑transcriptional regulation may involve specific microRNAs or RNA‑binding proteins that target the STAT3 3′ UTR, a hypothesis that warrants future investigation. Overall, our study uncovers a previously uncharacterized mode of STAT3 regulation by a natural compound and provides compelling experimental evidence linking traditional Chinese medicine-derived bufalin, ferroptosis suppression, and DKD progression, offering both conceptual advances and translational potential for clinical diagnosis and therapy.

## Supplementary Material

Supplemental Material

Supplementary_materials Clean.docx

## Data Availability

All data generated or analyzed during this study are available upon request from the corresponding author.

## Abbreviations

α-SMA: alpha-smooth muscle actin
ACSL4: acyl-CoA synthetase long-chain family member 4
CKD: chronic kidney disease
Col I: collagen I
DMSO: dimethyl sulfoxide
DKD: diabetic kidney disease
ECM: extracellular matrix
EMT: epithelial-mesenchymal transition
Fe^2+^: ferrous ion
Fer-1: ferrostatin-1
FN: fibronectin
Fe^2+^: ferrous ion
GPX4: glutathione peroxidase 4
GSH: glutathione
HG: high glucose
MDA: malondialdehyde
NC: negative control
RTECs: renal tubular epithelial cells
RT-qPCR: real-time quantitative polymerase chain reaction
SCr: serum creatinine
siRNA: small interfering RNA
SLC7A11: solute carrier family 7 member 11
STAT3: signal transducer and activator of transcription 3
TIF: tubulointerstitial fibrosis
