# Supplementary figures and images for: Bufalin post-transcriptionally suppresses STAT3 to alleviate renal ferroptosis and tubulointerstitial fibrosis in diabetic kidney disease
Source: Ren Fail. 2026 May 26;48(1):2667591. doi: 10.1080/0886022X.2026.2667591 (PMC13215403; doi:10.1080/0886022X.2026.2667591)

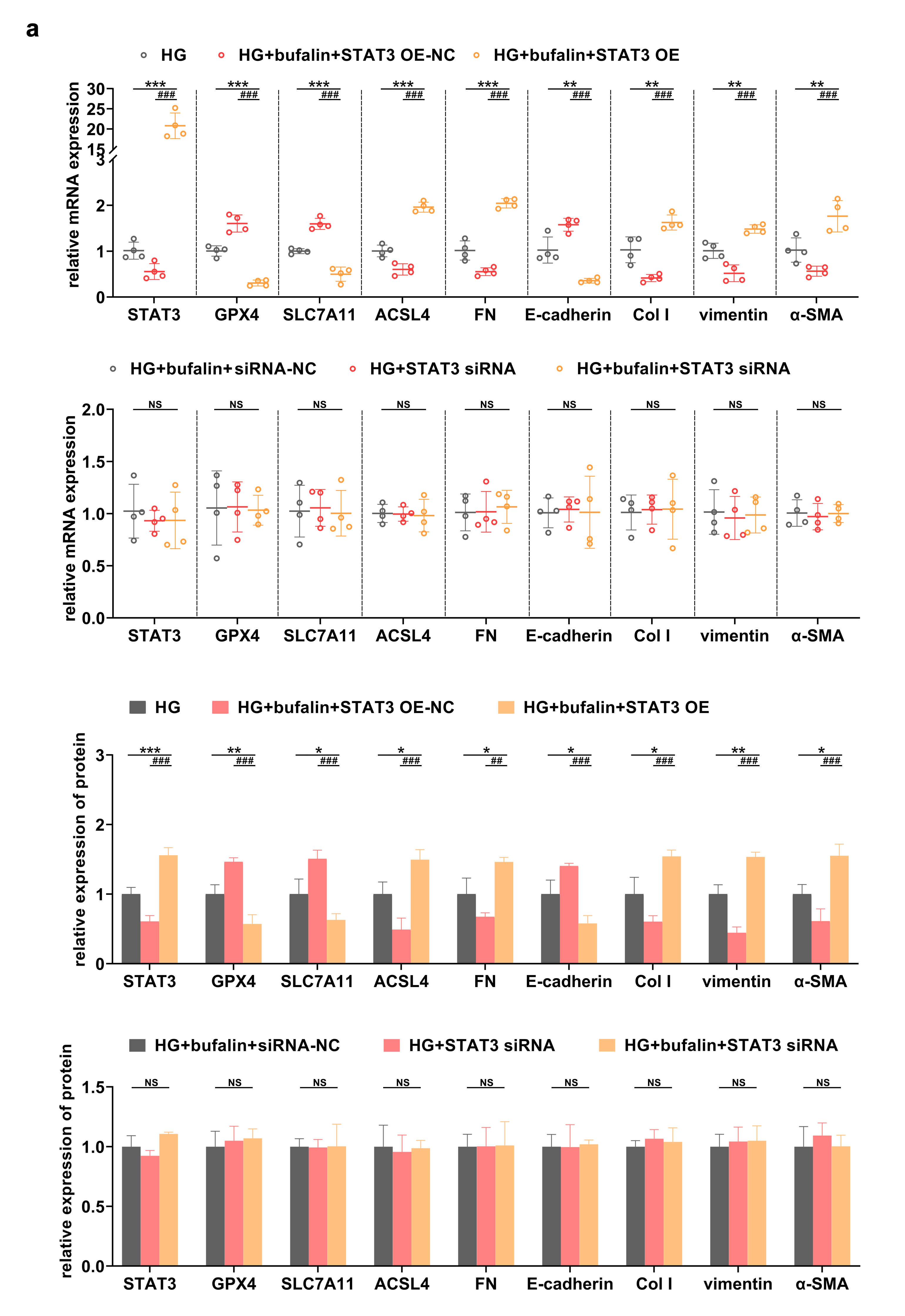

Supplement: Supplemental Material [file IRNF_A_2667591_SM5597.tif]
