# Supplementary material for: Bufalin post-transcriptionally suppresses STAT3 to alleviate renal ferroptosis and tubulointerstitial fibrosis in diabetic kidney disease
Source: Ren Fail. 2026 May 26;48(1):2667591. doi: 10.1080/0886022X.2026.2667591 (PMC13215403; doi:10.1080/0886022X.2026.2667591)
Supplement: Supplementary_materials Clean.docx [file IRNF_A_2667591_SM4261.docx]

**Supplementary table S1. Overview of the primer sequences for RT-qPCR.**

| Gene | Forward and reverse primers (5′ -3′) |
| --- | --- |
| fibronectin | F: GCAAGAAGGACAACCGAGGAAA  R: GGACATCAGTGAAGGAGCCAGA |
| collagen I | F: GTCAGACCTGTGTGTTCCCTACTCA  R: TCTCTCCAAACCAGACGTGTTC |
| E-cadherin | F: TCGGAAGACTCCCGATTCAAA  R: CGGACGAGGAAACTGGTCTC |
| vimentin | F: CGTCCACACGCACCTACAG  R: GGGGGATGAGGAATAGAGGCT |
| α-SMA | F: CAGCAAACAGGAATACGACGAA  R: AACCACGAGTAACAAATCAAAGC |
| GPX4 | F: ATACGCTGAGTGTGGTTTGC  R: CTTCATCCACTTCCACAGCG |
| SLC7A11 | F: CCTCTGACGATGGTGATGCTCTTC  R: GGTGCTGAATGGGTCCGAGTAAAG |
| ACSL4 | F: GGACTAGGACCGAAGGACACAT  R: GGAACAGCGGCCATAAGTGT |
| STAT3 | F: CACCTTGGATTGAGAGTCAAGAC  R: AGGAATCGGCTATATTGCTGGT |
| β-Actin | F: CATCCGTAAAGACCTCTATGCCAAC  R: ATGGAGCCACCGATCCACA |

Remarks: α-SMA, alpha-smooth muscle actin; GPX4, glutathione peroxidase 4; SLC7A11, solute carrier family 7 member 11; ACSL4, acyl-CoA synthetase long-chain family member 4; STAT3, [signal transducer and activator of transcription 3](https://www.ncbi.nlm.nih.gov/gene/6774)

**Supplementary table S2. Analysis of the target of bufalin and its correlation with ferroptosis**

| term | overlap | *P*-value | adj. *P*-value | odds ratio | combined score |
| --- | --- | --- | --- | --- | --- |
| ferroptosis | 32/1550 | 0.014521 | 0.053476 | 1.895881 | 8.023698 |
| ferroptosis_driver | 20/676 | 0.001399 | 0.008217 | 2.578725 | 16.948080 |
| ferroptosis_marker | 3/59 | 0.059873 | 0.148106 | 4.129589 | 11.627001 |
| ferroptosis_suppressor | 19/950 | 0.108284 | 0.212057 | 1.511797 | 3.3607183 |
| ferroptosis_unclassified | 5/142 | 0.063338 | 0.148845 | 2.709691 | 7.4767559 |

**Supplementary table S3. Network pharmacological analysis of bufalin and screening of DKD-related target molecules.**

| symbol | description | UniProt ID | GIFtS | GC id | score |
| --- | --- | --- | --- | --- | --- |
| PI4KB | phosphatidylinositol 4-kinase beta | Q9UBF8 | 58 | GC01M151291 | 1.38 |
| STAT3 | signal transducer and activator of transcription 3 | P40763 | 67 | GC17M042313 | 1.25 |
| TYK2 | tyrosine kinase 2 | P29597 | 66 | GC19M010350 | 0.94 |
| WEE1 | WEE1 G2 checkpoint kinase | P30291 | 61 | GC11P009573 | 0.88 |
| PTPN1 | protein tyrosine phosphatase non-receptor type 1 | P18031 | 63 | GC20P050510 | 0.83 |
| PFKFB3 | 6-phosphofructo-2-kinase/fructose-2,6-biphosphatase 3 | Q16875 | 56 | GC10P006144 | 0.7 |
| CYP17A1 | cytochrome P450 family 17 subfamily A member 1 | P05093 | 63 | GC10M102830 | 0.58 |
| KIF11 | kinesin family member 11 | P52732 | 61 | GC10P092574 | 0.58 |
| MC4R | melanocortin 4 receptor | P32245 | 59 | GC18M060371 | 0.49 |
| OPRM1 | opioid receptor Mu 1 | P35372 | 61 | GC06P174892 | 0.49 |
| EPHB4 | EPH receptor B4 | P54760 | 66 | GC07M106449 | 0.38 |
| INSR | insulin receptor | P06213 | 67 | GC19M007112 | 0.38 |
| MTOR | mechanistic target of rapamycin kinase | P42345 | 69 | GC01M011106 | 0.31 |
| PDE1C | phosphodiesterase 1C | Q14123 | 56 | GC07M031616 | 0.31 |

**Supplementary figure S1. Bufalin downregulates STAT3 via post-transcriptional regulation.**


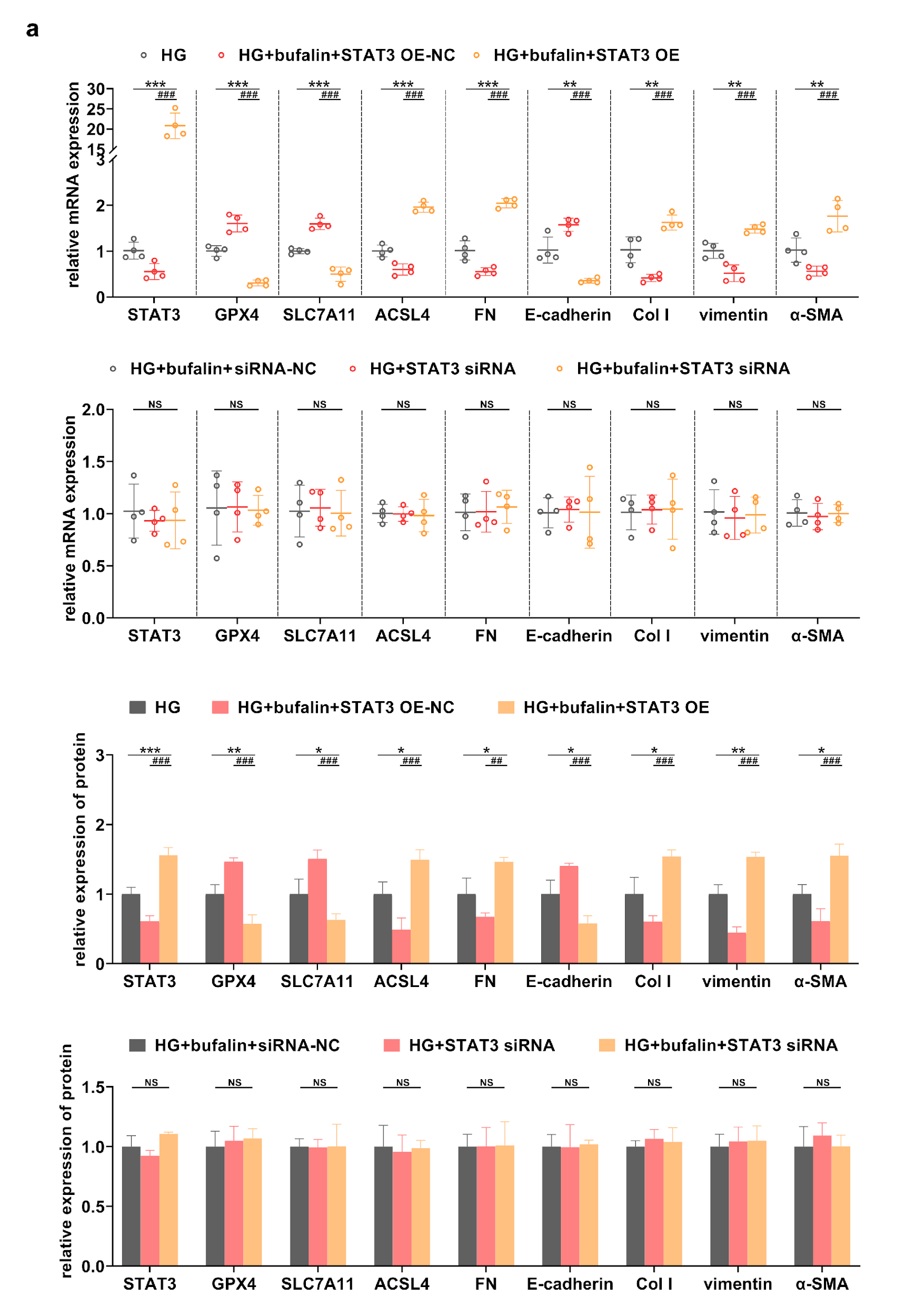


(a) In RTECs pretreated with bufalin, STAT3 siRNA or STAT3 overexpression plasmid were respectively transfected, and the mRNA and protein expression levels of ferroptosis and TIF-related indicators were detected by RT-qPCR and Western blot. The data are expressed as the mean ± SD. ^*^*P*<0.05, ^**^*P*<0.01, ^***^*P*<0.001, ^##^*P*<0.01, ^###^*P*<0.001 vs. the indicated group. NS, non-significant.
